# Children and adolescents with neurodevelopmental disorders show cognitive heterogeneity and require a person-centered approach

**DOI:** 10.1038/s41598-021-97551-6

**Published:** 2021-09-16

**Authors:** María Elena Márquez-Caraveo, Rocío Rodríguez-Valentín, Verónica Pérez-Barrón, Ruth Argelia Vázquez-Salas, José Carlos Sánchez-Ferrer, Filipa De Castro, Betania Allen-Leigh, Eduardo Lazcano-Ponce

**Affiliations:** 1 Children’s Psychiatric Hospital Juan N. Navarro, Av. San Fernando 86, Belisario Domínguez Secc 16, Tlalpan, 14080 Mexico City, Mexico; 2 Reproductive Health Division, Center for Population Health Research, National Institute of Public Health, Av. Universidad No. 655, Col. Santa María Ahuacatitlán, 62100 Cuernavaca, Morelos Mexico; 3 CONACYT, Center for Population Health Research, National Institute of Public Health, Cuernavaca, Morelos Mexico; 4 Center for Population Health Research, National Institute of Public Health, Cuernavaca, Morelos Mexico; 5 Academic Secretariat, School of Public Health of Mexico, National Institute of Public Health, Cuernavaca, Morelos Mexico; 6 Dirección de Salud Reproductiva, Centro de Investigaciones en Salud Poblacional, Instituto Nacional de Salud Pública, 7ª Cerrada de Fray Pedro de Gante 50, Col. Sección XVI, Tlalpan, 14000 Mexico City, Mexico

**Keywords:** Rehabilitation, Paediatric research, Diagnosis, Paediatric research, Disability, Neurological manifestations

## Abstract

We aimed to identify patterns of cognitive differences and characterize subgroups of Mexican children and adolescents with three neurodevelopmental disorders (NDD): intellectual disability (ID), autism spectrum disorders (ASD) and attention deficit/hyperactivity disorder (ADHD). The sample included 74 children and adolescents 6–15 years; 34% had ID, ASD or ADHD, 47% had ID in comorbidity with ASD, ADHD or both, 11% had ASD + ADHD, 8% were children without NDD. We applied WISC-IV, Autism Diagnostic Interview-Revised, Mini-International Neuropsychiatric Structured Interview, Child Behavior Checklist, and UNICEF Child Functioning Module. We evaluated the normality of the WISC-IV sub-scales using the Shapiro-Francia test, then conducted a latent class analysis and assessed inter-class differences in terms of household, parent and child characteristics. The following four-class solution best fit the data: “Lower Cognitive Profile” (LCP), “Lower Working Memory” (LWM), “Higher Working Memory” (HWM), “Higher Cognitive Profile” (HCP). LCP included most of the children with ID, who had a low Working Memory (WM) index score. LWM included mainly children with ASD or ID + ADHD; their Perceptual Reasoning (PR) and Processing Speed (PS) index scores were much higher than those for Verbal Comprehension (VC) and WM. HWM included children with ASD or ADHD; their scores for PR, PS and VC were high with lower WM (although higher than for LWM). HCP included children without NDD and with ASD or ADHD or both and had the highest scores on all indices. Children with NDD show cognitive heterogeneity and thus require individualized treatment plans.

## Introduction

Neurodevelopmental disorders such as Intellectual Disabilities (ID, DSM-5) or Disorders of Intellectual Development according to International Classification of Diseases-11 (ICD-11) nomenclature, Autism Spectrum Disorder (ASD) and Attention-Deficit/Hyperactivity Disorder (ADHD)^1^ have a high prevalence in childhood and constitute a large proportion of global disability^2^. ID is estimated to be higher in low- and middle-income countries^3^ and in 2016 while 2.7 million children with developmental disabilities (including ID, ASD and ADHD) lived in higher income countries, 50.2 million children with developmental disabilities lived in lower and middle-income countries^4^. Globally, ID prevalence has been estimated at around 1% (for 1980–2009)^3^ while more recently (2017) it was estimated at 3.2%^5^. In the US, ID has been estimated at 1.10%^6^ and although data on prevalence in Latin America are scarce^7^, in Mexico a 2015 national survey using a UNICEF questionnaire estimated 8.3% of Mexican children 5–17 years-old have socio-behavioral difficulties while 2% have multiple difficulties (cognitive, behavioral and physical)^8^. ASD prevalence worldwide has been estimated as ranging from 0.08 to 9.3%^9^, and prevalence in the US has been estimated between 1.3 and 2.6%^6,10^, while in Mexico data show a prevalence of 0.87%^11^. Finally, ADHD has a mean worldwide prevalence of 3.4% (2.6–4.5%) in children and adolescents < 18 years old^12^, 5.9% in youth and 2.5% in adults^13^ while in the US ADHD has been estimated at 9.04%^6^. In Mexico a recent study estimated an ADHD prevalence of 16% using a screening tool applied to a large school-based sample of 7–8 year olds^14^. Also, neurodevelopmental disorders (NDD) show high comorbidity^15^; for example, many children and adults with ASD also have ID, ranging from 26% in one study in Sweden^16^ to 33% and up to 53% in studies done in the US^17,18^. Some studies find that anywhere from 40 to 83% of children with ASD also have ADHD^19^ with other studies indicating 28–87% of children with ASD show symptoms of ADHD^20^. Other research has also found children with ID more commonly have ADHD^21^.

ID represents an atypical cognitive development^22^ and intelligence quotient (IQ) measurement has a strong tradition as a central element for ID diagnosis (IQ < 70). However, characterizing the specific cognitive dysfunction present is challenging since different cognitive impairments are aligned with similar IQs. Certain impairments can be more closely related to functional difficulties and behavioral problems^23^. Simultaneously, variability in IQ is one of the most salient dimensions of ASD heterogeneity^24^, although there is some consensus that more severe cognitive outcomes are observed in individuals with ID in comorbidity with ASD, as compared to those with only ID^25^. The cognitive dysfunction underlying these disorders is highly heterogeneous; furthermore, given that recent changes in medical classifications recognize that there is high comorbidity among them, great clinical and research challenges exist.

Cognitive performance of people with ASD has also been studied with measures of IQ. Some authors suggest that the Perceptual Reasoning Index of the Wechsler Adult Intelligence Scale-IV (WISC-IV) is the best intelligence estimate for individuals with ASD (instead of the full WISC-IV IQ scale)^26^. Many individuals with ASD + ADHD have a low Working Memory Index and, to a lesser degree, a low Processing Speed Index (both are WISC-IV subscales)^27,28^. Also, complexity increases when multiple sub-threshold neurodevelopmental symptoms occur^29^; sub-threshold ADHD symptoms may lead to impaired outcomes as often as when the individual has the full syndrome^30^. However, there are scarce findings regarding cognitive profiles of people with ID + ASD + ADHD^31^, in relation to other adaptive, behavioral and functioning/disability correlates.

Person-centered analysis, in contrast to variable-centered analysis, allows identification of groups of individuals taking into account their heterogeneous nature^32,33^. Latent class analysis^34^ allowed identifying unexpected differences among children, adolescents and adults (3–70 age range) in the Netherlands, with mild intellectual disability and borderline intellectual functioning. This analysis proposed a five class solution of subpopulations differentially related to personal or environmental variables, including those related to family and friends^35^. Another latent class analysis of children 4–17 years with ADHD and neurodevelopmental and mental health problems in USA households proposed a four-class solution with groups ranging from “low comorbidity” (most children, 64.5%) to “high comorbidity” (exhibiting the greatest impairments, the fewest children, 3%), while the group termed “predominantly developmental disorders” (almost 14% of children) showed predominantly comorbidity of ADHD + ID or ADHD + ASD^36^. Likewise, a latent profile analysis of a large national sample of children with ASD (although children who also had ID were under-represented in this study, as is common in ASD research literature) in North America, yielded five profiles among children 6–18 years who varied in terms of IQ, adaptive behavior, levels of aggression, anxiety, hyperactivity and behavioral rigidity^37^. A salient characteristic of ASD is its “unpredictable cognitive heterogeneity”^38^ and, as Nowell et al.^33^ suggest, the inclusion of variables beyond ASD symptoms, such as intellectual functioning scores, could help in the identification of subgroups through person-centered models. Among children in USA from 8 to 13 years, again using latent profile analysis, Dajani et al.^39^ identified differences in executive function in typical children and children with ASD, ADHD and both. A three class solution emerged named “above average” (33%); “average” (24%) and “impaired” (43%), the latter mostly children with ASD + ADHD. But, as far as the literature search we conducted, no study was found analyzing people with ID + ASD + ADHD in comorbidity, with person-center models.

Given the above, the objective of this research was to identify and characterize the cognitive heterogeneity of Mexican children and adolescents with NDD (ID, ASD, ADHD, or a comorbid diagnosis) using a person-centered analysis.

## Methods

### Participants and recruitment

**Figure 1:**
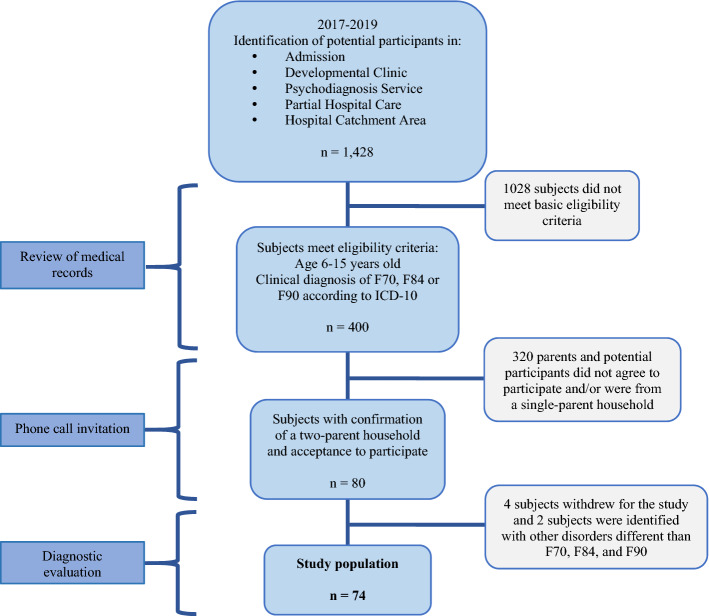
Identification of study population, children with and without neurodevelopmental disorders (F70-Mental Retardation, F84-Pervasive Developmental Disorders, F90-Hyperkinetic Disorder), Mexico City, Mexico.

The study population included 74 children and adolescents (Fig. 1). The inclusion criteria were children: (1) with a ID, ASD, or ADHD diagnosis based on the ICD-10 classification^40^, confirmed through testing by our research team staff; (2) 6–15 years; (3) both parents are alive and although not necessarily cohabitating, both are willing to participate in the diagnostic testing process and sign informed consent. The participants were recruited from current patients of the “Children’s Psychiatric Hospital Juan N. Navarro” with the exception of six participants referred by hospital personnel from among their social contacts (these last were also assessed to confirm they did not have a psychiatric diagnosis). Evaluations were performed after signed informed consent of parents and signed informed assent of children with appropriate development and with capacity to grant it. When any child seemed resistant to testing or distracted, testing was done on a subsequent visit. The instruments were applied during no more than four sessions that lasted 1–2 h each. During sessions, care was always taken to ensure participating children were feeling well and willing to participate, and children were given breaks during the testing process.

The study was carried out in accordance with the Declaration of Helsinki and all procedures were approved by the respective institutional ethics’ committees of the “Children’s Psychiatric Hospital Juan N. Navarro” and the National Institute of Public Health.

### Measures

The Wechsler Intelligence Scale for Children-Fourth version (WISC-IV)^41^ was used to diagnose ID. An IQ of less than 70 was defined as a diagnosis of ID, based on the ICD-11^40,42^. The WISC-IV includes four indices: Verbal Comprehension (VC), Perceptual Reasoning (PR), Working Memory (WM), and Processing Speed (PS). The psychometric properties of WISC-IV have been validated for Mexican children, and norms and standardization have been developed at the national level for various ages^41,43–45^. WISC-IV was applied by two psychologists specialized in cognitive assessments, each one with at least 10 years of experience.

Two additional psychologists, also with at least 10 years of experience, applied the Autism Diagnostic Interview-Revised (ADI-R)^46^, a semi-structured interview applied to the parents, to diagnose ASD. The ADI-R has been used extensively for diagnosis of ASD in Mexican children and meets DSM-IV and DSM-5 criteria better than other existing tools^11,47,48^. Also, diagnostic utility of this test has been established for Spanish-speaking Latino populations^49,50^. Inter-rater reliability for Mexican populations is 0.83–0.94 with internal consistence between 0.69 and 0.95^51–53^. The ADI-R algorithm generates scores for three domains of autism symptoms: reciprocal social interactions, communication (verbal and nonverbal), as well as restricted and repetitive stereotyped behaviors and interests, with the clinician also taking into account whether the disorder is evident by 36 months of age or before. Each domain has distinct cutoff points for an ASD diagnosis and elevated scores indicate problematic behavior in that domain; cut off scores are: reciprocal social interaction > 10, communication in children who are verbal > 8 and in nonverbal children > 7, while for restricted and repetitive stereotyped behaviors and interests > 3; for evidence of the disorder at 36 months or earlier the cutoff score is 1 (versus zero if there is no evidence at or before this age).

The Mini-International Neuropsychiatric Structured Interview version for children and adolescents (MINI Kid) was used to provide a categorical diagnosis of ADHD^51^. This interview was administered by a certified child psychiatrist with a PhD in psychology, as well as three residents in child psychiatry under her supervision, to parents in the presence of and where possible with participation by their child. This tool is based on the DSM-IV and ICD-10, has been validated in Spanish with a high inter-evaluator reliability of 0.9–1, test–retest reliability of 0.60–0.75 and concurrent validity with a clinical interview of 0.35–0.54^54–58^. A diagnosis of ADHD is given with a score of 6 out of 18. We also used the Child Behavior Checklist (CBCL)^59^ in order to provide a dimensional assessment of ADHD. Specifically, we used the attention problems subscale within the syndrome scale and the attention deficit/hyperactivity problems subscale from the DSM-oriented scales of the checklist. CBCL was self-administered with paper and pen by the parents. This tool has good internal consistency for the total problems scale (intra-class correlation coefficient = 0.97), externalizing problems scale (0.94), and for the internalizing problems scale (0.90)^60^. For the CBCL t-scores we used cutoff points of 65–69 to confirm a borderline diagnosis and above 69 to confirm a clinical diagnosis of ADHD.

We also used the 2017 version of the Child Functioning Module for children 5–17 years old developed by UNICEF and the Washington Group^61–63^. This module was applied by a psychologist to the parents to identify functioning difficulties in their child relating to 13 domains: seeing, hearing, walking, self-care, communication, learning, memory, concentration, accepting change, behavior regulation, making friends, anxiety, and depression. Those children with parental reports of “a lot of difficulty” or “total impossibility” in at least one domain were classified as having functioning difficulty, except for anxiety and depression for which “daily” was considered functional difficulty.

Moreover, we collected information about the child’s: age, sex, regular assistance to school, school type (public vs. private), education type (regular vs. special education), and current school level. Regarding parents we collected information on age, educational level and employment status. Mothers’ and fathers’ employment status was categorized as unpaid (housewives and unemployed) and paid occupation (any employment with a salary). We also registered socioeconomic level (low vs. middle) and household type (extended family, defined as the child or children, one or two parents, and other adult relatives, vs. nuclear family, the child or children and one or two parents but no other adult relatives).

### Data analysis

We estimated the prevalence, mean, and standard deviation of the household, parent, child, and clinical characteristics according to variable type. After validation of normality for the WISC-IV indices or sub-scales (WM, VC, PS, and PR) using the Shapiro-Francia test, we conducted a latent class analysis, seeking to identify subgroups of children (small clusters known as latent classes) within the study population, according to cognitive profiles.

To identify the appropriate number of classes we used Bayesian (BIC) and Akaike (AIC) criterion information, along with the log likelihood. For a latent class model, parameters estimated included marginal means, which may be thought of as the prevalence reflecting the likelihood that a cognitive characteristic exists in an individual, given membership in a class. Moreover, we assessed the inter-class differences in terms of household, parent, child, and clinical characteristics using Fisher’s exact test or analysis of variance (ANOVA), depending on the variable type. We also did Bonferroni post-hoc analysis to identify significant differences between classes. All analyses were performed using STATA 15.0 (Stata Corporation. Texas: College Station, 2015).

### Ethics approval

This research project was approved by the ethics committees of the Children’s Psychiatric Hospital Juan N. Navarro and the National Institute of Public Health of Mexico and was therefore performed in accordance with the ethical standards laid down in the 1964 Declaration of Helsinki and its later amendments.

### Consent to participate

Signed informed consent of all parents was obtained as well as signed informed assent of children with appropriate development and capacity to grant it.

### Consent for publication

All authors consent to the publication of this manuscript.

## Results

### Study population characteristics

Child, parent, and household characteristics are presented in Table 1. Average child age was 9.67 years old (± 2.78 SD), most were males (79.73%), who reported regular attendance to school (94.59%) mainly to public (74.32%), regular (not special education) (72.97%), and elementary schools (70.27%). Their mothers were on average 38.67 years old (± 6.90 SD), most had a high school (36.49%) or junior-high (29.73%) education level and over half reported an unpaid occupation (54.05%). Meanwhile, their fathers were on average 40.99 years old (± 7.58 SD), had a junior high-school education (37.84%) or high school education level (31.08%), and almost all reported paid occupation (95.95%). Most of the children lived in nuclear (not extended) families (75.68%) and slightly more than half had a low socioeconomic level (54.05%).

**Table 1:** Study population characteristics: child, parent, household, and clinical variables, Psychiatric Children's Hospital, Mexico City, Mexico, 2017–2019 (n = 74).

Characteristics	n	%	Mean	± SD
**Child**				
Age	74		9.67	± 2.78
Sex				
Male	59	79.73		
Female	15	20.27		
Regular school attendance				
No	4	5.41		
Yes	70	94.59		
School type				
Public	55	74.32		
Private	15	20.27		
Education type				
Regular	54	72.97		
Special education	16	21.62		
Current level in school				
None	3	4.05		
Pre-school	3	4.05		
Elementary school	52	70.27		
Junior high school	15	20.27		
High school	1	1.35		
**Parent**				
Mother's age	74		38.67	± 6.90
Mother's educational level^a^				
Elementary school or less	8	10.81		
Junior-high school	22	29.73		
High school or some university	27	36.49		
University	17	22.97		
Mother's employment status^b^				
Unpaid occupation	40	54.05		
Paid occupation	34	45.95		
Father's age	74		40.99	± 7.58
Father's educational level^a^				
Elementary school or less	8	10.81		
Junior-high school	28	37.84		
High school or some university	23	31.08		
University	15	20.27		
Father's employment status^b^				
Unpaid occupation	3	4.05		
Paid occupation	71	95.95		
**Household**				
Household type				
Extended	18	24.32		
Nuclear	56	75.68		
Socio-economic level^c^				
Low	40	54.05		
Middle	28	37.84		
**Child’s clinical variables**				
Cognitive profile (WISC-IV Index Score)				
Working memory (WM)	74		0.93	± 1.27
Verbal comprehension (VC)	74		1.08	± 1.40
Processing speed (PS)	74		1.50	± 1.41
Perceptual reasoning (PR)	74		1.70	± 1.57
Single or comorbid neurodevelopmental diagnoses				
Intellectual disability (ID)	5	6.76		
Attention deficit hyperactivity disorder (ADHD)	10	13.51		
Autism spectrum disorders (ASD)	10	13.51		
ID + ADHD	11	14.86		
ID + ASD	14	18.92		
ADHD + ASD	8	10.81		
ID + ADHD + ASD	10	13.51		
Participants without neurodevelopmental disorders	6	8.11		
Functioning difficulties (Washington Group-UNICEF Module)^d^				
Without functioning difficulty	18	24.32		
With functioning difficulties	56	75.68		

^a^Mother's or Father's education level: Elementary school or less (Incomplete or complete elementary school), Junior-high school (Incomplete or complete junior-high school), High school or some university (Complete high-school or incomplete bachelor's degree), and University or more (Complete Bachelors degree or postgraduate level).

^b^Mother's and Father's employment status: Unpaid (Housewife or unemployed), paid occupation (any salaried employment).

^c^Six missing values for socioeconomic level.

^d^Functioning difficulties according to the Washington Group-UNICEF module in at least one of the following domains: seeing, hearing, walking, self-care, communication, learning, memory, concentration, acceptance of change, behavior regulation, making friends, anxiety and depression.

For the WISC-IV index scores, Table 1 shows that children had the lowest scores for the WM index (0.93 ± 1.27), followed by the VC (1.08 ± 1.40) and PS indices (1.50 ± 1.41), while children had the highest scores for the PR index (1.70 ± 1.57). In terms of neurodevelopmental diagnoses, fewer children (6.76%) had ID than those with ASD or ADHD (13.51% each). Somewhat higher percentages of children had ID + ASD (18.92%); ID + ADHD (14.86%); or ID + ASD + ADHD (13.51%). A slightly lower percentage of children had ASD + ADHD (10.81%). Finally, 75.68% of the children exhibited functioning difficulty according to the Washington Group-UNICEF measurement tool.

Table 2 presents the incremental fit statistics and likelihood for the best class solution. The five-class solution had an increase in the Bayesian and Akaike values, after a consistent decrease among lower numbers of classes. Therefore, the four-class solution was selected as the best fitting model.

**Table 2:** Incremental fit statistics and log likelihood for best class solution, latent class analysis, Psychiatric Children's Hospital, Mexico City, Mexico, 2017–2019 (n = 74).

Class solution	Log likelihood (LL)	Akaike Information Criterion (AIC)	Bayesian Information Criterion (BIC)
1 class	− 519.52	1055.05	1073.48
2 classes	− 421.59	869.18	899.14
3 classes	− 398.38	830.76	869.93
4 classes	**− 376.85**	**799.70**	**852.69**
5 classes	− 376.85	803.70	861.3

Bold values indicate the 4 class solution was the best fitting model.

### Class description

**Figure 2:**
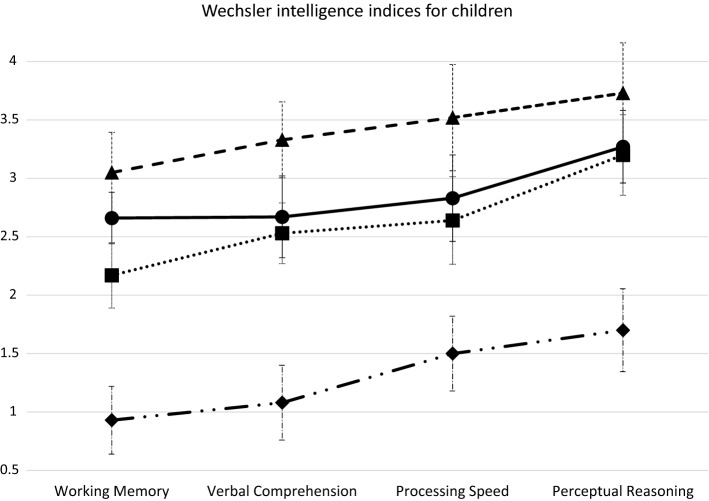
Marginal means of Wechsler intelligence indices associated with latent class membership, children with and without neurodevelopmental disorders, Mexico City, Mexico.

Figure 2 depicts the four-class profiles associated with class membership. Class 1, which can be described as the “Lower Cognitive Profile” (LCP) subgroup, showed lower marginal means across all the WISC-IV indices. Classes 2 and 3 showed similar marginal means across four indices with differences mainly in WM; therefore, these classes can be described as the “Lower Working Memory” (LWM) and “Higher Working Memory” (HWM) subgroups, respectively. Meanwhile, Class 4 showed a higher marginal means across all indices, and thus corresponds to a “Higher Cognitive Profile” (HCP) subgroup.

Table 3 presents the prevalence of socio-demographic characteristics, neurodevelopmental diagnoses, and functioning difficulties, as well as mean WISC-IV indices scores across the four classes. The LCP group (class 1) had a higher number of children (n = 33), 78.79% boys, 90.91% attended school regularly, mostly public school (90%), 43.33% attended special education and 69.70% were in elementary school. This class had the lowest scores for all WISC-IV indices as compared to the other classes. Within this class, the lowest scores were for the WM index (0.06 ± 0.35) and the highest for the PS index (0.27 ± 0.57). In this class, more children had ID + ASD (36.4%), while many had all three disorders studied (ID + ASD + ADHD, 27.27%), a fourth had ID + ADHD (24.24%) and 12.13% had only ID. This class has the largest proportion of children with functioning difficulties (90.91%, p = 0.03).

**Table 3:** Comparison of child, parent, household, and clinical variables across the four latent classes, Psychiatric Children's Hospital, Mexico City, Mexico, 2017–2019 (n = 74).

Characteristics	Latent classes				p value
	Class 1—“Lower Cognitive Profile” (n = 33)	Class 2—“Lower Working Memory” (n = 12)	Class 3—“Higher Working Memory” (n = 17)	Class 4—“Higher Cognitive Profile” (n = 12)	
**Child**					
Age (Mean ± SD)	9.55 ± 2.68	10.42 ± 3.45	9.35 ± 2.47	9.75 ± 2.96	0.77**
Sex (%)					
Male	78.79	66.67	100.00	66.67	0.04*
Female	21.21	33.33	0.00	33.33	
Regular school attendance (%)					
No	9.09	0.00	5.88	0.00	0.81*
Yes	90.91	100.00	94.12	100.00	
School type (%)					
Public	90.00	83.33	68.75	58.33	0.09*
Private	10.00	16.67	31.25	41.67	
Education type (%)					
Regular	56.67	91.67	93.75	91.67	0.01*
Special education	43.33	8.33	6.25	8.33	
Current level in school (%)					
None	9.09	0.00	0.00	0.00	0.51*
Pre-school	9.09	0.00	0.00	0.00	
Elementary school	69.70	66.67	70.59	75.00	
Junior high school	12.12	25.00	29.41	25.00	
High school	0.00	8.33	0.00	0.00	
**Parent**					
Mother's age (Mean ± SD)	38.61 ± 7.51	38.58 ± 5.30	38.65 ± 7.30	39.00 ± 6.81	0.99**
Mother's educational level^a^ (%)					
Elementary school or less	12.12	8.33	11.76	8.33	0.32*
Junior-high school	33.33	41.67	35.29	0.00	
High school or some university	33.33	33.33	23.53	66.67	
University	21.21	16.67	29.41	25.00	
Mother's employment status^b^ (%)					
Unpaid occupation	69.70	50.00	47.06	25.00	0.05*
Paid occupation	30.30	50.00	52.94	75.00	
Father's age (Mean ± SD)	39.82 ± 7.27	40.50 ± 4.87	42.59 ± 8.49	42.42 ± 9.39	0.58**
Father's educational level^a^ (%)					
Elementary school or less	12.12	25.00	5.88	0.00	0.43*
Junior-high school	42.42	41.67	41.18	16.67	
High school or some university	27.27	16.67	29.41	58.33	
University	18.18	16.67	23.53	25.00	
Father's employment status^b^ (%)					
Unpaid occupation	3.03	8.33	5.88	0.00	0.86*
Paid occupation	96.97	91.67	94.12	100.00	
**Household**					
Household type (%)					
Extended	27.27	16.67	17.65	33.33	0.71*
Nuclear	72.73	83.33	82.35	66.67	
Socioeconomic level^c^ (%)					
Low	60.61	75.00	58.82	16.67	0.15*
Middle	39.39	25.00	41.18	83.33	
**Child's Cognitive profile, Neurodevelopmental Diagnoses and Functioning**					
WISC-IV Indices Scores (mean ± SD)					
Working memory^d^	0.06 ± 0.35	0.17 ± 0.39	1.65 ± 0.86	3.08 ± 0.66	< 0.01**
Verbal comprehension^d^	0.06 ± 0.24	0.25 ± 0.45	2.06 ± 0.75	3.33 ± 0.98	< 0.01**
Processing speed^e^	0.27 ± 0.57	2.08 ± 0.67	2.06 ± 0.82	3.50 ± 1.00	< 0.01**
Perceptual reasoning^e^	0.24 ± 0.50	2.25 ± 1.14	2.71 ± 0.59	3.75 ± 0.96	< 0.01**
Single or comorbid neurodevelopmental diagnoses (%)					
Intellectual Development Disorder (ID)	12.13	8.33	0.00	0.00	< 0.01*
Attention Deficit Hyperactivity Disorder (ADHD)	0.00	8.33	41.18	16.67	
Autism Spectrum Disorders (ASD)	0.00	25.00	23.53	25.00	
ID + ADHD	24.24	25.00	0.00	0.00	
ID + ASD	36.36	16.67	0.00	0.00	
ADHD + ASD	0.00	8.33	35.29	8.33	
ID + ADHD + ASD	27.27	8.33	0.00	0.00	
Participants without neurodevelopmental disorders	0.00	0.00	0.00	50.00	
Functioning difficulties^f^ (%)					
Without functioning difficulty	9.09	33.33	35.29	41.67	0.03*
With functioning difficulty	90.91	66.67	64.71	58.33	

^a^Mother's or Father's educational level: elementary school or less (Incomplete or complete elementary school), Junior-high school (Incomplete or complete junior-high school), high school or some university (Complete high-school or incomplete bachelor's degree), and University or more (Bachelor's degree or postgraduate level).

^b^Mother's and Father's employment status: unpaid (Housewives and unemployed), and paid occupation (any employment with salary for the realized work).

^c^6 missing values for socioeconomic level.

^d^Bonferroni post hoc analysis: no significant differences were observed between class 1 and class 2; significant differences were observed between the rest of the pairs of classes.

^e^Bonferroni post hoc analysis: no significant differences were observed between class 2 and class 3; significant differences were observed between the rest of the pairs of classes.

^f^Functioning difficulties according to the Washington Group-UNICEF module in at least one of the following domains: seeing, hearing, walking, self-care, communication, learning, memory, concentration, acceptance of change, behavior regulation, making friends, anxiety and depression.

*Exact Fisher Test.

**One factor ANOVA test.

The LWM group (class 2) (n = 12) had the highest mean age (10.42 ± 3.45), 66.7% boys, all these children attend school, mostly public school (83.33%) while 8.33% attended special education. This class also had a low score for the WM index (0.17 ± 0.39), with a slightly higher score for VC (0.25 ± 0.45). In this group of children, the PS (2.08 ± 0.67) and PR (2.25 ± 1.14) scores were much higher than the two previous indices. This class was more heterogeneous in terms of comorbidity, with the same prevalence of children with ASD (25.00%) or children with ID + ADHD (25.00%). Within the LWM class there were fewer children with ID + ASD (16.67%), as well as a prevalence of 8.33% for each of the following outcomes (ID or ADHD, ADHD + ASD, or ID + ADHD + ASD). Two-thirds of this group of children had functioning difficulties (66.67%).

The HWM group (class 3) (n = 17) included only boys, 94.12% attend school with two-thirds attending public school and only 6.25% receive special education. The WM index scores for this group of children is lower than those for the other WISC-IV indices, as occurs in the other classes. These children have virtually the same scores for the VC and PS indices (2.06 ± 0.75 and 2.06 ± 0.82, respectively). Finally, among these children the PR index has the highest value (2.71 ± 0.59). This class showed a higher prevalence of children with ADHD (41.18%), ADHD + ASD (35.29%), as well as a prevalence of 23.53% for children with ASD. About two-thirds of this group of children, as with the previous class, had functioning difficulties.

Finally, in the HCP group (class 4) (n = 12), two-thirds are boys, all attend school regularly, 41.67% of these children attend private school (more than in other classes), with 8.33% receiving special education. The cognitive functioning of this group of children is better than the children in the other classes, with higher scores in all WISC-IV indices. These children show the same ascending trend in WISC-IV index scores as the other classes, with the lowest for WM and the highest for PR. This class included all children without neurodevelopment disorders (50.0%), children with ASD (25.00%), ADHD (16.67%) or ASD + ADHD in comorbidity (8.33%). Slightly over half of these children showed functioning difficulties (58.33%).

### Class comparison

The only socio-demographic characteristics with statistically significant differences between classes were sex (p = 0.04) and education type (regular vs. special, p = 0.01), as well as a marginal difference in paid occupation among the mothers (p = 0.05). There were statistically significant differences between the WISC-IV indices scores for all classes (< 0.01). The Bonferroni post hoc analysis indicates that there were no significant differences between class 1 and 2 for the WM and VC indices; significant differences for these indices did exist between the rest of the possible class combinations. This analysis also showed there were no significant differences between classes 2 and 3 for the PS and PR indices; thus, significant differences for these indices were observed between the rest of the possible class combinations (see footnotes in Table 3).

In all classes the WM index scores were the lowest WISC-IV index. The PR index scores were the highest for all classes except the LCP class. For the WM, VC and PR indices there was a significant mean increase across the four classes; that is, each class had a higher mean score for these three indices than the previous class (Table 3). In the LWM and HWM classes there were differences precisely between the WM index scores (0.17 ± 0.39 vs. 1.65 ± 0.86) and also between these children’s VC index scores (0.25 ± 0.45 vs. 2.06 ± 0.75).

Only the LCP and LWM classes included children with ID, (alone or in combination with other diagnoses), with more in the former class. Children with ASD, ADHD or ASD + ADHD were grouped in classes 2, 3 and 4 (LWM and HWM as well as HCP). The only comorbidity that children in the HWM and HCP classes had is ASD + ADHD.

Moreover, we identified a significant decrease (p = 0.03) in prevalence of functioning difficulties across classes using the Washington Group-UNICEF measurement tool. That is, children in the LCP class had the highest prevalence of functioning difficulties while children in the HCP class had the lowest (Table 3).

## Discussion

This study used latent class analysis, a person-centered methodological strategy, of WISC-IV index scores to show the heterogeneity of the cognitive profiles of a group of Mexican children and adolescents, most of whom had neurodevelopmental disorders. All four profiles (classes) identified included children with different diagnoses and one class included typically developing children as well as children with NDDs.

This is one of the few latent class analyses that focuses on children with ID^24,35^. All the children with ID (alone or in comorbidity with other neurodevelopmental disorders) were in the two classes with the lowest cognitive scores: these were the “Lower Cognitive Profile” (LCP) and the “Lower Working Memory” (LWM) classes. In Toffalini et al.’s analysis of three different methods to estimate WISC scores in children with ID while aiming to avoid floor effects, WM scores were consistently the lowest and PS the next lowest, in comparison with VC or PR^64^. In our findings, the two classes that included all the children with ID showed low levels of WM and VC, with higher PS and PR scores, probably because our sample included a large proportion of children with moderate ID. Also according to Toffalini et al., the VC index tends to be lower in children with moderate ID as compared to those with mild ID^64^. Most of the children in our sample with ID + ASD were included in the LCP group but some were also in the LWM class. The low WM and VC scores we found in these groups of children coincides with Mungkhetklang et al. who report that in a sample of adolescents with a clinical diagnosis of ID or ID + ASD, the WM index was the lowest index for both groups and although verbal abilities were poor for all the participants, the adolescents with ID + ASD had the lowest scores^25^. The higher PS scores in these two classes of children in our study is consistent with Mungkhetklang et al. and the WISC-IV Manual, which suggests that children with ID show higher scores for PS than VC and PR^25,41^. Charman et al., found, as we did, lower VC scores for the WISC-III test in a group of children with ASD (ICD-10 Research Criteria), half of whom also had ID^65^. Our analysis also contributes to the description of children with ID + ASD, who are more impaired and have long been under-represented in the literature. Most studies focus on children with ASD who have an IQ above 85, thereby excluding children with ASD + ID. Moreover, as Tager-Flusberg and Kasari say, there is an especially significant “dearth of knowledge” about children with ID + ASD who are nonverbal^66^. More research is needed on children with this comorbidity, especially those who are nonverbal. Our study also provides data on children with the ID + ASD + ADHD triad, a comorbidity that to our knowledge has not been studied in terms of cognitive function. This is in spite of the fact that high levels of inattention and hyperactivity/impulsive behaviors are exhibited in children with NDD compared to their neurotypical peers^31^. Our analysis grouped most children with ID + ASD + ADHD (and also with ID + ASD) in the LCP class, with lower cognitive scores in general. This complex comorbidity (ID + ASD + ADHD) has clinical relevance because, as Gillberg et al. suggest, children presenting at an early age for a diagnosis tend to be the most impaired and may have ASD with multiple comorbidities but are often only diagnosed with ASD initially^67^. This is also a relevant clinical issue since the forthcoming ICD-11 describes six types of ASD with different cognitive levels, guiding clinicians and researchers to recognize, measure, and in general have greater awareness of cognitive heterogeneity among children with ASD (ICD-11).

The addition of the term “spectrum” to the ASD diagnosis was visionary (coined by Wing in the 90s)^68^ but at present autism is a diagnostic category that faces criticism on the grounds that it is a heterogeneous neurodevelopmental atypicality, and thus is sometimes expressed as “several autisms” whose symptoms underlie different etiologies. ASD sometimes includes other behaviors labeled as ADHD or anxiety disorders, among others. This is another example of why studying dimensions that cross diagnostic boundaries can be especially useful^69^. In our analysis, children with ASD were distributed in three classes, even though there were significant differences between these groups in terms of the cognitive index scores. While VC indices varied little between the groups, PS and PR scores showed significant differences. A possible explanation of low VC scores is that ours was a clinical sample and most children with pure ASD had a borderline IQ score, with only a few children in the normal IQ range. These low VC scores are consistent with Charman et al. who suggest that in clinical samples deficient verbal skills (which would result in lower VC Index scores) might be the reason for referral of many children with ASD^65^. Meanwhile, the children’s higher PS and PR scores indicates a cognitive profile similar to Klopper et al.’s findings, suggesting two phenotypic subgroups of children with ASD in which the severely socially impaired group showed the largest cognitive difficulties (as compared with the moderately socially impaired group)^70^. Nader et al. also found higher PR scores in children with ASD^28^.

Other studies have also used WISC-IV profiles to explore symptomatology and outcomes of children with ADHD and found as we did lower scores for the WM or PS indices specifically in these children^28,71–74^. Some authors argue that children with ASD + ADHD in comorbidity are more impaired than those with only one of these diagnoses, using an “additive” comorbidity perspective to explain these cognitive outcomes^28,39,75^. Our results correspond with Dajani et al.’s latent profile analysis of executive functions including WISC-IV in typically developing children, children with ASD, ADHD or ASD + ADHD, which suggest that classes based on executive function did not reproduce diagnostic categories^39^. These authors evaluated executive functions including WISC-IV indices and found a three class solution (“above average”, “average” and “impaired”) documenting the dimensional nature of executive functions across children (classes did not show distinct patterns of strengths or weakness neither did they reproduce diagnostic categories). The “average” and “impaired” groups included a mix of children with different diagnoses, and typical children fell into the “above average” but also the “average” profiles, suggesting heterogeneity of executive function abilities in children with NDD as well as in typical developing children. Despite this, 92% of children with ASD + ADHD were in the “impaired” group in comparison to 47% of children with ASD and 63% with ADHD; this has treatment implications because not all children with ASD or with ADHD need executive function interventions.

The identification of four cognitive profiles of subgroups of children, independent of their diagnoses, moves our findings towards a dimensional perspective more akin to psychopathology than to a categorical approach typical of more traditional psychiatric classification systems. The US National Institute of Mental Health (NIMH) Research Domain Criteria (RDoC) framework for classification of psychopathology encompasses neurobiology, observable behavior and aims to link brain circuits to specific behaviors and symptoms instead of focusing on traditional diagnostic categories. It involves different levels of analysis that focus on dimensional constructs (for example, fear, attention, memory) underlying core symptoms of mental disorders. These constructs do not have a one-to-one relationship with a diagnostic category^76^. That our four cognitive profiles did not correspond to diagnostic categories might imply that WISC-IV indices are level-specific dimensional constructs underlying NDDs and also typical development.

Musser and Raiker, in an integrated perspective stemming from developmental psychopathology and the RDoC domains of cognition, specifically working memory and positive valance (reward anticipation/delay/receipt), challenge ADHD as a categorical diagnosis. They emphasize the continuous distribution of ADHD symptoms in the general population as well as comorbid diagnosis and symptoms^77^. The authors highlight other research findings that suggest the lack of specificity of WM impairment in ADHD given the fact that it is also observed in association with inattention and impulsivity in typically developing youth (Tillman et al.^78^ as cited by Musser and Raiker)^77^. This idea is also in agreement in a recent transdiagnostic pediatric study evaluating diagnostic associations and a composite measure of PS^79^; Krammer et al. documented that PS was associated with reading, math and ADHD disabilities but not with ASD, if inattention is taken into account. Interestingly, the authors propose that PS should be a specific construct in transdiagnostic research frameworks since PS is a cognitive domain not currently included in the RDoC cognitive system.

It is well established that children with ASD tend to have good levels of PR^80^; nonetheless we found high PR scores in all the subgroups of children in this analysis (and thus, in children with various diagnoses as well as in typically developing children) except the children in class 1 (which included mostly children with ID alone or in comorbidity). Clark et al.^80^ recently documented that PR is a mediator between attention and math proficiency in children with ASD + ID or ASD + ADHD (as well as other neurodevelopmental conditions), suggesting that diagnostic classification did not necessarily influence the relationship between PR and other cognitive abilities, whether in children with ASD or with other neurodevelopmental conditions.

The NIMH RDoC constitutes a critique of the (previously predominant) psychiatric diagnostic classification system with a central idea of not simply labeling individuals, and in a sense force people’s characteristics to fit that label. By addressing meaningful psychopathological behaviors independent of a diagnostic category, it constitutes a framework that seeks to enhance knowledge of underlying mechanisms and processes contributing to personalized medicine or care^81^. Nonetheless, in order to apply it to infant psychopathology, NDDs and typical development in the earliest stages of life, there is still a long way to go in the development of translational measures clinically relevant to transdiagnostic approaches. For example, in terms of attentional disruptions, practice still largely relies on assessments via parental questionnaires or invasive methods; therefore, new measures are needed^81^. Certainly, there are common cognitive and biological processes across phenotypes (DSM5). For example, impairments in WM associated with inattention and impulsivity constitute a continuum in children with NDDs as well as typically developing youth, an issue raised by Musser^77^. It is also relevant to study samples of children with varied comorbidities in order to establish the role of these constructs in expression and potential remission over time of NDDs.

It has been over 10 years since the proposal by Insel and the NIMH that the scientific community transform the traditional psychiatric classification of diseases, emphasizing the dimension of neurobiology and observable behavior as a research framework. Casey et al.^76^ propose to “extend and enrich” the RDoC framework with a neurodevelopmental perspective including the recognition of the following issues: (a) observing developmental trajectories across time to understand atypical as well as typical development; (b) taking into account sensitive periods when experiences can have a greater impact on development and (c) dynamic interaction of systems (“developmental cascades”) between differentially maturing brain systems and developmental time, as well as their interaction with environment and context. Musser and Raiker’s review on ADHD proposes an integrated approach of the RDoC system and developmental psychopathology; this is a subdiscipline of contemporary models of development that assumes reciprocal interaction of biological, psychological and social systems to explain both typical and atypical development and favors a dimensional approach^77^. Recently, Talbott and Miller proposed recommendations for future research in the ASD field from a transdiagnostic perspective and noted that this approach has rarely been applied to childhood psychopathology and NDDs in infancy^81^. These authors suggest that this approach allows identification of processes shared across disorders and that underlie and maintain symptoms, developing an integrated model of transdiagnostic assessment and intervention for infants with prodromal risk signs to allow early intervention.

Traditional treatment based on categorical models, even when they reach children with NDDs at early stages (a challenge especially for children living in LMICs), focuses on symptoms that have already manifested when they are also already causing individual, familial and social negative impact. A true preventive effort from a transdiagnostic, dimensional perspective that identifies key early indicators before full-blown symptoms emerge is essential to NDDs intervention. Additionally, if mechanisms in the transition from risk to disorder which are shared across NDDs can be identified through this type of perspective they can then be targeted by prevention programs that may have a greater positive impact that targeting more specific factors, indicators or symptoms^81^. These pre-behavioral markers of risk may reveal unprecedented treatments and therapeutics to apply transdiagnostically^82^.

Applying dimensional impairment perspectives in treatment may contribute to more effective interventions since, as is indicated by our findings, not all children with ASD or ADHD are likely to need the same cognitive interventions. Accordingly, treatments must be differentiated and dimensional; some of these dimensional candidates for targeted intervention include WM, PS and PR. For example PS is an important factor in attentional deficits, academic achievement and even peer relationships (Thorsen 83 as cited by Kramer)^79^; thus it may be productive to provide PS enhancing interventions to some children not based on diagnosis but based on their PS performance. Given that some studies have found PR to be a mediator between attention and math skills and that it is not moderated by diagnosis^80^,^81^.

### Strengths and limitations

For an adequate interpretation of these results, some methodological aspects need to be considered. Even though our study is cross-sectional, it is one of the relatively few studies that evaluate neurodevelopmental disorders, both alone and in comorbidity; thus this analysis seeks to form part of a new research agenda that takes a “dimensional/overlap approach to neurodevelopmental disorders”^70^. However, recent conceptualizations refer to NDD as disorders with “cluster comorbidity”^15^. Classifications such as the DSM-5 have begun to include comorbidity only recently and there is a dearth of scientific evidence in this area, which is a strength of our study.

This study is based on a relatively small clinical sample of children. However, we used measures that have been standardized or validated not only in higher-income countries but in Mexico and other middle- and lower-income countries as well. We also used a series of measures to evaluate ID, ASD and ADHD; this allowed us to evaluate hyperactivity and impulsivity symptoms, which are challenging for differential diagnosis^67^. We also studied children with the diagnostic triad of ID + ASD + ADHD. Another strength of our study is that we studied children from a middle-income country and whose parents had a variety of educational and socio-economic levels. This is important since only a fraction (2.3%) of articles published about infancy are based on data from low- or middle-income countries, in spite of the fact that that is where 90% of infants live worldwide^84^. In addition, although a higher proportion of children with NDD live in low- and middle-income countries such as Mexico, there is a dearth of published studies on these disorders based on studies that are carried out in these parts of the world^3^.

Another limitation is the floor effect in measurement of cognitive profiles using WISC-IV^25,85–88^. This could decrease the range and variability of the results. However, we consider this to be an acceptable limitation given that this is the only instrument that measures IQ that has been standardized and evaluated in Mexican children^89^. Additionally, the WISC-IV that we used is reported to have greater sensitivity to ADHD than other Wechsler versions^90^. In addition, another strength was that we did diagnostic and cognitive measurements simultaneously.

Also, the Washington Group-UNICEF tool for measuring functioning difficulties is designed for use at the population level, for large surveys, and not for clinical diagnosis^61^. Nevertheless, we used this measure to provide additional information about the children in the sample and not to diagnose a specific disorder or mental health issue.

## Conclusions

Our analysis used a person-centered approach, which allowed us to provide an evidence base about cognitive heterogeneity in children with a different neurodevelopmental disorders. Furthermore, our results provide a warning against the use of a wide diagnostic umbrella and the detrimental effects of clinical preconceptions about patients. These results support the need for diagnosis and intervention in NDDs that provide individualized, targeted treatment plans, taking into account the specific needs of patients from a dimensional, transdiagnostic approach.

## Data Availability

The data cannot be made publicly available given that the informed consent forms stated that “the information you provide for this study will be confidential and will be used only by the research project team and will not be available for any other purpose”.
